# Primacy of theory? Exploring perspectives on validity in conceptual psychometrics

**DOI:** 10.3389/fpsyg.2024.1383622

**Published:** 2024-05-30

**Authors:** Josh Joseph Ramminger, Niklas Jacobs

**Affiliations:** ^1^Faculty of Life Sciences, Department of Psychology, Humboldt University of Berlin, Berlin, Germany; ^2^Department of Psychology, Philipps-University, Marburg, Germany

**Keywords:** validity, theory, conceptual psychometrics, philosophy of science, metatheory, methodology

## Abstract

Several conceptions of validity have emphasized the contingency of validity on theory. Here we revisit several contributions to the discourse on the concept of validity, which we consider particularly influential or insightful. Despite differences in metatheory, both Cronbach and Meehl’s construct validity, and Borsboom, Mellenbergh and van Heerden’s early concept of validity regard validity as a criterion for successful measurement and thus, as crucial for the soundness of psychological science. Others, such as Borgstede and Eggert, regard recourses to validity as an appeal to an (unscientific) folk psychology. Instead, they advocate theory-based measurement. It will be demonstrated that these divergent positions converge in their view of psychological theory as indispensable for the soundness of psychological measurement. However, the formulation of the concept (and scope) of scientific theory differs across the presented conceptions of validity. These differences can be at least partially attributed to three disparities in metatheoretical and methodological stances. The first concerns the question of the structure of scientific theories. The second concerns the question of psychology’s subject matter. The third regards whether, and if, to which extent, correlations can be indicative of causality and therefore point toward validity. These results indicate that metatheory may help to structure the discourse on the concept of validity by revealing the contingencies the concrete positions rely on.

## Introduction

How shall we understand the concept of validity? Which methodological implications arise from conceptions and critique of validity? These questions have been subject to a lively discourse. Within this discourse, substantial divergence regarding metatheory and methodology in psychology is present (see Cronbach and Meehl, 1955; Messick, 1989; Slaney, 2017; Borsboom, 2023). For us, metatheory deals with the investigation of scientific theories, as well as their relation to stances in theory of science. In our view, philosophy and the sciences can particularly benefit from the investigation of the logical connection between metatheory and methodology (see Hanfstingl, 2019; Uher, 2023). Validity, as one domain of disagreement, is commonly understood to address whether one measures what is intended to be measured. However, this definition has been criticized because it presupposes that one *is* measuring something and that that which shall be measured is measurable (Michell, 2009, 11–33). For some validity concerns the soundness of a conclusion drawn from a measurement outcome (see Markus and Borsboom, 2013). One of us has argued elsewhere that the validity debate is a prime example of a philosophical-psychological discourse, as in it logical connections between metatheory and methodology are illustrated (Ramminger, 2023).

One such logical connection is *scientific theory*. Philosophy of science investigates the structure of scientific theories (e.g., Balzer et al., 1987). Metatheoretical assumptions can structure scientific theories because scientific theories can deal with the same entities based on the same empirical evidence and still be different (Ramminger et al., 2023). Concrete (i.e., clearly defined) scientific theories are furthermore an important element of the working scientists’ epistemic processes (Hastings et al., 2020).

However, scientometric studies show that not all psychological research can be regarded as theory-driven (McPhetres et al., 2021; Wendt and Wolfradt, 2022), even though low replication rates in psychology have repeatedly been attributed to deficiencies in theory-building and application (Fiedler, 2017; Muthukrishna and Henrich, 2019; Oberauer and Lewandowsky, 2019; Green, 2021; Witte, 2022; Ramminger et al., 2023). Such agreement lacks regarding the relationship between theory and validity, even though validity is a prerequisite for replicability (Flake et al., 2022). For example, Borgstede (2019) has argued that some applied validity research is atheoretical. In addition, different theory-based conceptions of validity differ in their concept of scientific theories (Borsboom et al., 2004; Buntins et al., 2017). Furthermore, even when adhering to one specified conception of validity (such as Cronbach and Meehl’s construct validity), the underlying theory (i.e., the nomological net) is not always stated explicitly (for an introduction see Ziegler et al., 2013).

In what follows, we will show that different conceptions of validity and validity’s relation to scientific theory stem from metatheoretical assumptions. These differences concern the structure of scientific theories, the question of psychology’s subject matter (Wendt and Funke, 2022; Wendler and Ramminger, 2023), as well as methodological considerations (e.g., *whether, and if, to which extent* correlations can be indicative for causality and therefore pointing toward validity). Finally, we will show that proponents and critics of the employment of validity converge in their assumption that *theory-basedness* is at least necessary to ensure the soundness of psychological measurement.

## Metatheory, validity, and scientific theory

Several conceptions of validity can be traced back to their metatheoretical assumptions. Some movements in philosophy of science have therefore been associated with conceptions of validity. Examples range from descriptive empiricism, in the case of criterion validity, to logical positivism and scientific realism, in the case of construct validity (see Markus and Borsboom, 2013, 5–14; Slaney, 2017).^1^ Furthermore, the semantic view of scientific theories (Balzer et al., 1987) is part of Borgstede and Eggert’s account of theory-based measurement (Borgstede and Eggert, 2023). Our aim is not to settle questions in philosophy of science, but to demonstrate that different conceptions of validity converge in their assumption that validity is contingent upon theory. Moreover that this convergence of positions is present despite the divergent philosophies of science to which the positions adhere.

Different metatheoretical assumptions commonly entail a view on the nature of psychological attributes. Psychometricians often conceptualize their object of measurement as an unobservable mental construct (and consistently apply latent variable modeling). However, Borgstede and Eggert (2023) tend toward a behaviorist’s perspective, thus seeing behavior as the crucial subject matter of psychology. Borsboom (2023) speaks of psychological attributes as *organizing principles* and thus adheres to network psychometrics and advocates for the rehabilitation of content validity. We are concerned here with the question of how authors of different perspectives in theory of science approach the relationship between validity and theory. We will present three positions associated with individual authors more in-depth, Cronbach and Meehl (1955), Borsboom’s early perspective (Borsboom et al., 2004), and Borgstede and Eggert’s (2023) position which rejects the term validity altogether but is still concerned with ensuring that psychologists know what they measure.

These accounts were selected due to several factors. Cronbach and Meehl (1955) developed construct validity, arguably the conception of validity most utilized in contemporary psychology, for example it is largely adopted by the *Standards for Educational and Psychological Testing* (American Educational Research Association, American Psychological Association, and National Council on Measurement in Education, 2014). Consequently, the other accounts engage with Cronbach and Meehl (1955), while Borgstede and Eggert (2023) also address Borsboom et al. (2004). Furthermore, the term validity either denotes a characteristic of tests or test score interpretations (Borsboom et al., 2003b; Borsboom and Markus, 2013). Two selected papers (Borsboom et al., 2004; Borgstede and Eggert, 2023) engage with the first meaning, while Cronbach and Meehl (1955) aim to address the second one. The selected stances diverge in philosophical questions (e.g., realism or how scientific theories shall be structured), however one can logically infer from these approaches, that validity must be theory based. This convergence—despite philosophical divergence - is thus a strong argument for the necessity of theory for validity. Lastly, Borgstede and Eggert (2023) developed their approach analogous to measurement and theory building in the natural sciences whose methodological rigor is an ideal often adhered to in psychology (see James, 1892; Wieczorek et al., 2021).

First, we turn to *construct validity* (Cronbach and Meehl, 1955). As noted above, construct validity was associated with several traditions in philosophy. Since we aim to demonstrate that several accounts of validity are influenced by metatheoretical stances, more specifically traditions in philosophy of science and that these accounts align in their emphasis on the importance of scientific theory, we do not settle the question whether construct validity is indeed contingent to logical positivism (as Borsboom et al., 2004 argue) or scientific realism (Rozeboom, 1984; Slaney, 2012; see also Slaney, 2017). However, since two of the three positions we address *definitely* reject logical positivism (see Borsboom et al., 2004; Borgstede, 2022), we focus here on a logical positivist’s interpretation of construct validity (for an introduction to logical positivism see Creath, 2023) to stretch the logical space and show that validity conceptions resting on logical positivism likewise regard a well-formulated theory as a prerequisite for the investigation of a measurement instrument’s validity.^2^

Such an account of construct validity emphasises that (a) Cronbach and Meehl insisted that the nomological network gives constructs their meaning (by making the relations of the constructs explicit) and that (b) Cronbach and Meehl are especially concerned with cases in which at least one variable studied cannot be regarded as observable, i.e., they are interested in the relation of theoretical constructs to observables. For example, if you were to create a conscientiousness personality test item based on this account, you would *a priori* point out expected relations (i.e., a high correlation with average punctuality). After a first test phase of the item, you would either confirm this expectation, concluding that you measured conscientiousness or, in case you found an unexpected correlation, conclude that you did not measure conscientiousness/create a new hypothesis that conscientiousness in fact does not correlate highly with punctuality (see Cronbach and Meehl, 1955).

Consistently, according to Borgstede, the positivist assumes the task of science to be translating observations into theory-language to determine the truth of the theoretical propositions. This practice would be contingent on a syntactic conception of scientific theories. The syntactic view regards a scientific theory as a system of propositions. These syntactic structures are identified by applying the theory to empirical relational structures through operationalization, or correspondence rules as they are called in theory of science (Borgstede, 2022, 18–19). Therefore, the relation between observables and non-observables is a central element of construct validity *and* positivism.

The importance of scientific theory in determining construct validity can be further demonstrated by Cronbach and Meehl’s assertion that the “types of evidence” for construct validity depend “on the theory surrounding the construct” (Cronbach and Meehl, 1955, 288). Such types of evidence could be factor analyses, another one correlations. Moreover, the execution of a measurement may result in two potential outcomes: either concluding that the results indicate construct validity or adjustment of the nomological net, consequently impacting the underlying theory. Thus, construct validity is judged after measurement.

Borsboom and several colleagues, the second position we review more in-depth, disagree with the metatheoretical stances of a positivist’s reading of construct validity. In their early work, Borsboom and several colleagues advocate for a validity concept based on a realist’s metatheory (Borsboom et al., 2004, Borsboom et al., 2009). For Borsboom, Mellenbergh, and van Heerden, logical positivism and its application to validity theory rests on the possibility of making meaningful statements without referring to existing attributes.

For the logical positivist, advocating for construct validity, a test could be regarded as valid for measuring a construct if the empirical relations between test scores match the theoretical relations between constructs. That theorist would continue to argue that the meaning of psychological constructs is determined via the relation of the corresponding concepts in a nomological network. In contrast, Borsboom et al. adhere to a realist account of validity, since they regard it as inconceivable, “how the sentences *Test X measures the attitude toward nuclear energy* and *Attitudes do not exist* can both be true” (Borsboom et al., 2004, 1063). Their commitments to philosophical realism (see also Borsboom et al., 2003a; Borsboom, 2005, 6–8; Borsboom also quotes Hacking, 1983 and Devitt, 1991 when introducing realism) allow Borsboom and colleagues to infer two crucial methodological implications. First, they regard a test as being “valid for measuring an attribute if and only if (a) the attribute exists and (b) variations in the attribute causally produce variations in the outcomes of the measurement procedure” (Borsboom et al., 2004, 1061). Secondly, a theory about the response behavior of people is necessary, otherwise, validity judgements cannot be made. In other words, if the attribute causes variations in the test scores, this causal influence must occur somewhere in the process of responding itself and theories have to take this response process into account.^3^

To better understand this approach, we can again refer to our example of the conscientiousness item. Following Borsboom’s and colleagues’ 2004 approach, you would establish a theory of the causal role of conscientiousness for the response given to the item. For example, conscientious people will read the item carefully and unveil an ambiguity, which evokes an answer divergent from non-conscientious people.

How can one test this theory? One could infer that the answers given by divergent subgroups which are expected to be very conscientious (potentially air traffic controllers) differ from the answers given by groups that are expected to be less conscientious (potentially graphic designers, this example only has illustrative purpose). Note that this represents a test of the underlying theory, not the validity of the conscientiousness item.

The question of validity thus becomes the question whether the attribute of interest exists and how that attribute—this is where the theory comes in—causally affects test scores.

Furthermore, Borsboom et al. (2004) criticize correlation-based and anti-realist positions approaches to validity, since two absurd conclusions would follow from them. Firstly that two highly correlated constructs are identical (see also Borgstede’s (2019) critique), and secondly that when measuring a group of objects that do not show variation in the interesting attribute, it would become *a priori* impossible to conclude that the measurement is valid since for a variance of zero the correlation is undefined.^4^ Suppose one wants to measure the length of rods using a meter stick and that all rods have the same length. One could not conclude that the meter stick is a valid measure of length (see Borsboom et al., 2004).

Finally, Borsboom et al. (2004) emphasis on ontology in validity leads them to critique positions that regard validity to be judged *after measurement,* since knowledge of the nature of the object of measurement would imply knowledge of the steps one has to take to measure that object. Thus, validity would become an *a priori* matter of metatheory (ontology) and scientific theory. Ontology deals with condition (a), the existence of the attribute, and scientific theory with condition (b), whether ‘variations in the attribute causally produce variations in the outcomes of the measurement procedure’.

As a third perspective, Borgstede and several colleagues do not agree that the attribute necessarily exists (Buntins et al., 2017). They claim that the central problem of psychological measurement is not unobservability, but the lack of well-defined concepts. Like Borsboom and colleagues, they explicitly reject logical positivism and the associated *syntactic* view of scientific theories (Borgstede and Eggert, 2023). Borgstede and Eggert follow the *semantic* view in theory of science, according to which a *substantive* fundamental principle structures a scientific theory (Borgstede, 2022; Borgstede and Eggert, 2023; for an philosophical introduction see Balzer et al., 1987). For Borgstede, one such principle might be behavioral selection (Borgstede, 2022, 31). Since behavior is observable, the problem of psychological concepts for Borgstede and Eggert is not that they are observable or latent, but that they are *poorly defined* (Borgstede and Eggert, 2023).

According to Borgstede and Eggert, one cannot determine whether one measures what one wants to measure independently of a measurement theory. When using the operational theory of measurement (see Stevens, 1946) one (by definition) measures what one intends to measure, since there is no difference between what is to be measured and the indicator. The representational measurement theory (see Krantz et al., 1971), however, gives testable criteria for investigating whether one is measuring what one wants to measure (Buntins et al., 2017).

Consistently, for Borgstede and Eggert, the problem with psychological concepts is that they are rarely defined within the framework of a substantive (formal) theory (Borgstede and Eggert, 2023). In this context, a substantive (and) formal theory can be described as a hierarchical network. Substantively, this network is structured by a *fundamental* underlying principle (e.g., behavioral selection) and more *specific* principles (e.g., specific types of reinforcement) that explain empirical phenomena (e.g., change in behavior). These principles are formally defined (Borgstede and Eggert, 2023). This often implies a mathematical definition, but one can also find formalizations that utilize formal logic (Buntins et al., 2015). In psychology, descriptively speaking, validity would commonly term “the degree to which the variable measured by a test corresponds to concepts of everyday language” (Buntins et al., 2017). However, if validity is supposed to anchor psychological concepts in common-sense, which trivially is not mathematically accurate, then it is not possible to measure in a theory-based way.

Their proposed antidote is theory-based measurement, which they regard as necessary and sufficient for *knowing what we are measuring*. That is because proper theory informs us about the steps necessary to measure the entities the theory entails. Put differently, the knowledge of the measurement procedure stems from the theorized relation between the objects of measurement (observable phenomena). For example, we can adhere to Newton’s second law to measure mass, since it allows us to use a beam scale (Borgstede and Eggert, 2023, for an application to behavioral measurement see Borgstede and Anselme, 2024).

How would this approach relate to our running example of the construction of a conscientiousness item? This is a puzzling question, and one could even argue that here we face the danger of a category error. It is tempting to understand conscientiousness as a mental attribute, in which case it would not be straightforward to align the concept of conscientiousness with Borgstede’s behaviorist leanings.

Borgstede suggests behavioral selection as a fundamental principle which can structure psychological theories. The content of these theories should be the interaction of individuals and their environment. Therefore, conscientiousness possibly needs a redefinition regarding its causal relation to the fundamental principle and the other entities postulated in the general theory. Drawing on Borgstede’s exemplary fundamental principle of behavioral selection, one would have to relate conscientiousness to it and the less abstract entities and principles in the theory net. Such a relation could draw on principles of social interaction in early human societies, which could potentially contribute to the explanation of the genesis of conscientiousness from natural selection. Another possibility is that such a theory would not include entities that correspond to concepts that are derived from common language. In this case, one may conclude that conscientiousness does not exist.

Comparing the three discussed accounts of validity and their relation to theory, several aspects deserve additional emphasis. Although Borgstede and Eggert reject the recourse to validity in the sense the term is often used in psychology, they still regard theory as necessary to solve the epistemic questions the validity discourse raises. Logically, Borsboom et al. (2004) are concerned with something akin, since they reject the idea that one can determine whether one has measured what one wanted to measure *after* the measurement procedure (unlike Cronbach and Meehl). Since theories describe causal processes, Borsboom et al. (2004), as well as Borgstede and Eggert (2023), Borgstede (2019) converge in the assumption that determining validity implies that we need to adhere to an *a priori* theory of the *causal* properties of our variable of interest. Thus, they stand in stark opposition to Cronbach and Meehl’s approach of judging construct validity *a posteriori* (possibly based on correlations, which are viewed as indicative of causality). All three positions presented formulate their idea of psychological measurement, to which its construct is known, within the context of a philosophy of science and attribute central relevance to scientific theories.

## Conclusion and limitations

All three positions align in emphasizing the central relevance of scientific theory in understanding and defining validity in psychological measurement. They all underscore the importance of having a well-formulated theoretical framework when considering the validity of measurement instruments in psychology. However, they differ in their specific philosophical and metatheoretical assumptions, as well as in the question of whether validity is judged *a priori* or *a posteriori*. Consistently, two of the formulated approaches reject the inference of validity from empirical results (e.g., correlation matrices), since they adhere to measurement procedures derived by *a priori* reflections on the causal properties of the variable under investigation. They thus emphasize that validity conclusions are justified by adherence to theoretical propositions. Of course, the quality of validity concepts depends on metatheoretical criteria such as consistency. Furthermore, the question of the feasibility of the methodological implications in research projects is also highly relevant (see also Borsboom, 2023). However, questions about the criteria of measurability (e.g., Michell, 1999; Markus and Borsboom, 2012) and the potential context dependency of validity (see for a critique, Larroulet Philippi, 2021) exceed the scope of this paper. After all, in this essay, we were concerned precisely with the inner, logical, relationship of metatheory and methodology in the discourse on validity. Logically, all three positions engage with the conditions of knowing what psychologists are measuring (*a priori* or *a posteriori*), therefore Borgstede and Eggert are part of this discourse, even though they reject the term (and a certain notion of) validity. This paper demonstrates the interconnectedness of metatheory, theory, and measurement and aims to encourage an appreciation of theory for the soundness of psychological measurement, which is not always present in contemporary psychometrics.

## Data availability statement

The original contributions presented in the study are included in the article/supplementary material, further inquiries can be directed to the corresponding authors.

## Author contributions

JR: Conceptualization, Investigation, Methodology, Project administration, Writing – original draft, Writing – review & editing. NJ: Conceptualization, Funding acquisition, Investigation, Methodology, Writing – review & editing.

## Footnotes

^1^Some scholars argue that logical positivism and empiricism should be used as a synonym (*cf.* Uebel, 2013). Markus and Borsboom (2013, 5–14) distinguish different forms of empiricism and relate these with different approaches to validity. We stick with their taxonomy for the purpose of differentiating between different metatheoretical foundations for validity concepts.

^2^However, it must be noted that since 1955 construct validity has evolved and that Cronbach in his later work regarded it as problematic to formulate the idea of construct validity in the language of positivism (Cronbach, 1989, 159; see also Slaney, 2017).

^3^It is not our intention to assert that Borsboom and the several colleagues, with which he put forth this conception still adhere to this position. As we have briefly touched upon, Borsboom recently elaborated on validity in network psychometrics and the implications of this approach for the ontology of psychological attributes (Borsboom, 2023). However, the early work with which we engage here is customized for latent variable analysis, which is still widely applied in psychometrics.

^4^This can be derived from *r*=￼ $\frac{\mathrm{cov}\left(x,y\right)}{\sigma_{x}\sigma_{y}}$ since one variance being zero (*x* or *y*) leads to a division by zero, which is undefined.
